# Identification of key genes and pathways for melanoma in the TRIM family

**DOI:** 10.1002/cam4.3545

**Published:** 2020-10-28

**Authors:** YiJun Xia, Jun Zhao, Chunjun Yang

**Affiliations:** ^1^ Department of Plastic and Reconstructive Surgery The Second Affiliated Hospital of Anhui Medical University Hefei Anhui Province China; ^2^ Department of Dermatology The Second Affiliated Hospital of Anhui Medical University Hefei Anhui Province China

**Keywords:** GEPIA, melanoma, Oncomine, prognosis, TRIM

## Abstract

Certain members of the TRIM family have been shown to have abnormal expression and prognostic value in cancer. However, in the development and progression of melanoma, the role of different TRIM family members remains unknown. To address this issue, this study used the Oncomine, UCSC, Human Protein Atlas, DAVID, and GEPIA databases to study the role of TRIMs in the prognosis of melanoma. Differential expression of TRIM2, TRIM7, TRIM8, TRIM18 (MID1), TRIM19 (PML), TRIM27, and TRIM29 may play an important role in the development of melanoma. The expression TRIM7 and TRIM29 appeared to be helpful in the identification of primary tumors and metastases. Survival analysis suggested that the expression of TRIM27 significantly affected the overall survival and disease‐free survival of melanoma, and its expression was confirmed by qRT‐PCR. Our results indicated that the expression level of TRIM27 might be a prognostic marker of melanoma.

## Graphical abstract

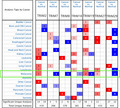

## 1 INTRODUCTION

As one of the malignant cutaneous tumors, melanoma is caused by malignant transformation of epidermal melanocytes. Melanoma ranks third of all malignant skin tumors, and its morbidity and mortality rates have risen steadily in the past decade.^1^ Melanoma is a disease that involves multistep, multifactorial regulation of complex signal transduction.^2^, ^3^, ^4^ The molecular mechanism of its development has been widely studied. The tripartite motif‐containing (TRIM) proteins regulate the occurrence, invasion, and apoptosis of certain tumors. TRIM2, TRIM28, TRIM29, TRIM59, and other TRIM family are relevant to malignancy of neurologic tumors such as glioma and digestive tumors such as gastric cancer, colorectal cancer.^5^, ^6^, ^7^, ^8^ From the N‐terminal to the C‐terminal, the protein of TRIM family has three typical domains: RING‐finger domain, B‐box domain, and helix‐helix domain.^9^, ^10^, ^11^, ^12^

To date, TRIM protein has been suggested to be part of cell proliferation and division, regulate cell metabolism and autophagocytosis, participate in chromatin modification and gene transcription, and be involved in tumor cell stemness and innate immunity.^13^, ^14^, ^15^, ^16^ In signaling pathways such as NF‐κB, TGF‐β, and PI3K/Akt, the TRIM family mediates the cell stemness of tumors and their ability to self‐renew through excessive activation or abnormal signals of these pathways.^17^, ^18^, ^19^, ^20^

Currently, some TRIM proteins have been reported to have prognostic value and abnormal expression in cancer. Nevertheless, there has been no reported association between the TRIM family and the prognosis and occurrence of melanoma. In this paper, we explored the relationship between TRIM family and melanoma by observing the expression of different TRIMs in melanoma patients and their relationship to clinical parameters.

## 2 MATERIALS AND METHODS

### 2.1 Differential expression gene screening

The TRIM family is a large gene family that has been found in more than 80 species, including humans. In the Oncomine database, we compared the levels of transcription of the TRIM family in cancerous and normal samples and screened for differentially expressed TRIM family members (https://www.oncomine.org/).^21^ The Oncomine is a database containing 715 microarray data sets that provide gene expression information in a variety of cancerous and noncancerous samples. A *p*‐value of 1E‐4 was considered as the screening cut‐off criteria and the top 10% genes were screened. Gene Expression Profiling Interactive Analysis (GEPIA) is a web portal of RNA sequencing data from GTEx and TCGA projects, which can perform single‐gene analysis, multi‐gene analysis, prognostic analysis, and profiling plotting in a variety of tumors. The mRNA expression patterns of screened TRIM family members were further measured in GEPIA.^22^

### 2.2 KEGG and GO enrichment analyses

The Database for Annotation, Visualization, and Integrated Discovery (DAVID) (https://david.ncifcrf.gov) was used to perform Gene Ontology (GO) and Kyoto Encyclopedia of Genes and Genomes (KEGG) enrichment analyses to elucidate the function, biological process, and enrichment pathway of the selected genes.^23^, ^24^, ^25^

### 2.3 Immunohistochemical analysis

After mRNA expression was verified, the screened genes were explored for protein expression in melanoma and normal skin tissues in the Human Protein Atlas database (http://www.proteinatlas.org).^26^ The Human Protein Atlas (HPA) contains nearly 20 common types of cancer based on immunohistochemical expression of the data, through which multiple genes in melanoma‐specific protein expression patterns can be identified.

### 2.4 Correlation analysis

After analyzing mRNA expression and protein expression patterns in melanoma patients, we performed three aspects of correlation analysis. As a gene family, we performed the correlation analysis between the selected key genes. Afterward, we searched the UCSC database (https://xenabrowser.net) to obtain the patients’ clinicopathological data and mRNA expression profiles, and to identify the relationship between TRIMs and the clinical parameters of melanoma patients.^27^ The UCSC database focuses on human and mouse genomes for visualization, comparison, analysis, and sharing of publicly available and user‐generated genome data sets. Moreover, The Tumor Immunological Estimation Resource (TIMER) platform (https://cistrome.shinyapps.io/timer/) was explored the correlation between tumor immune‐infiltrating cells (TIICs) and the TRIMs, and the statistical results were based on Spearman correlation analysis.^28^

### 2.5 Prognostic value of TRIMs in melanoma

The GEPIA was evaluated to investigate the prognostic significance of TRIM2, TRIM7, TRIM8, TRIM18 (MID1), TRIM19 (PML), TRIM27, and TRIM29 in melanoma, with overall survival (OS) and disease‐free survival (DFS) as evaluation indicators. Genes with *p* < 0.05 were considered to have prognostic value, based on the Kaplan‐Meier (KM) method. The effects of different TRIM expressions and clinicopathological characteristics on the OS of patients were implemented through Cox hazards regression analysis, and R software was implemented to complete statistical analysis (version 3.4.1).

### 2.6 RNA extraction and quantitative real‐time polymerase chain reaction (qRT‐PCR) analysis

Melanoma cell line A375 and the normal cell line HaCat were inoculated in a 6‐well plate culture for 24 h, and intracellular RNA was extracted by the TRIzol method (Invitrogen), and then complementary DNA was synthesized. The experiments were performed in the SYBR Premix Ex Taq kit (Takara Bio) protocol using glyceraldehyde‐3 phosphate dehydrogenase (GAPDH) expression as a reference. The oligonucleotide primers for TRIM18, TRIM27, TRIM29, and GAPDH are provided in Table 1. The relative expression of the TRIMs was measured using the 2^−△△CT^ method.

**TABLE 1:** Primer sequences used for qRT‐PCR amplification.

Primer	5′>3′
GAPDH	GACAGTCAGCCGCATCTTCT
	ACCAAATCCGTTGACTCCGA
TRIM18	CTGACCTGCCCTATTTGTCTG
	GCACAGTGTGATACTAGGATGC
TRIM27	AGCCCATGATGCTCGACTG
	GGGCACGACACGTTAGTCT
TRIM29	CTGTTCGCGGGCAATGAGT
	TGCCTTCCATAGAGTCCATGC

### 2.7 Gene enrichment analyses of prognostic TRIMs

Through the LinkedOmics database, TRIM27 was analyzed for Gene Set Enrichment Analysis (GSEA) to find its biological functions and associated inhibition or activation pathways (http://www.linkedomics.org/). A false discovery rate (FDR) <0.05 suggested GSEA with significant difference.

## 3 RESULTS

**FIGURE 1:**
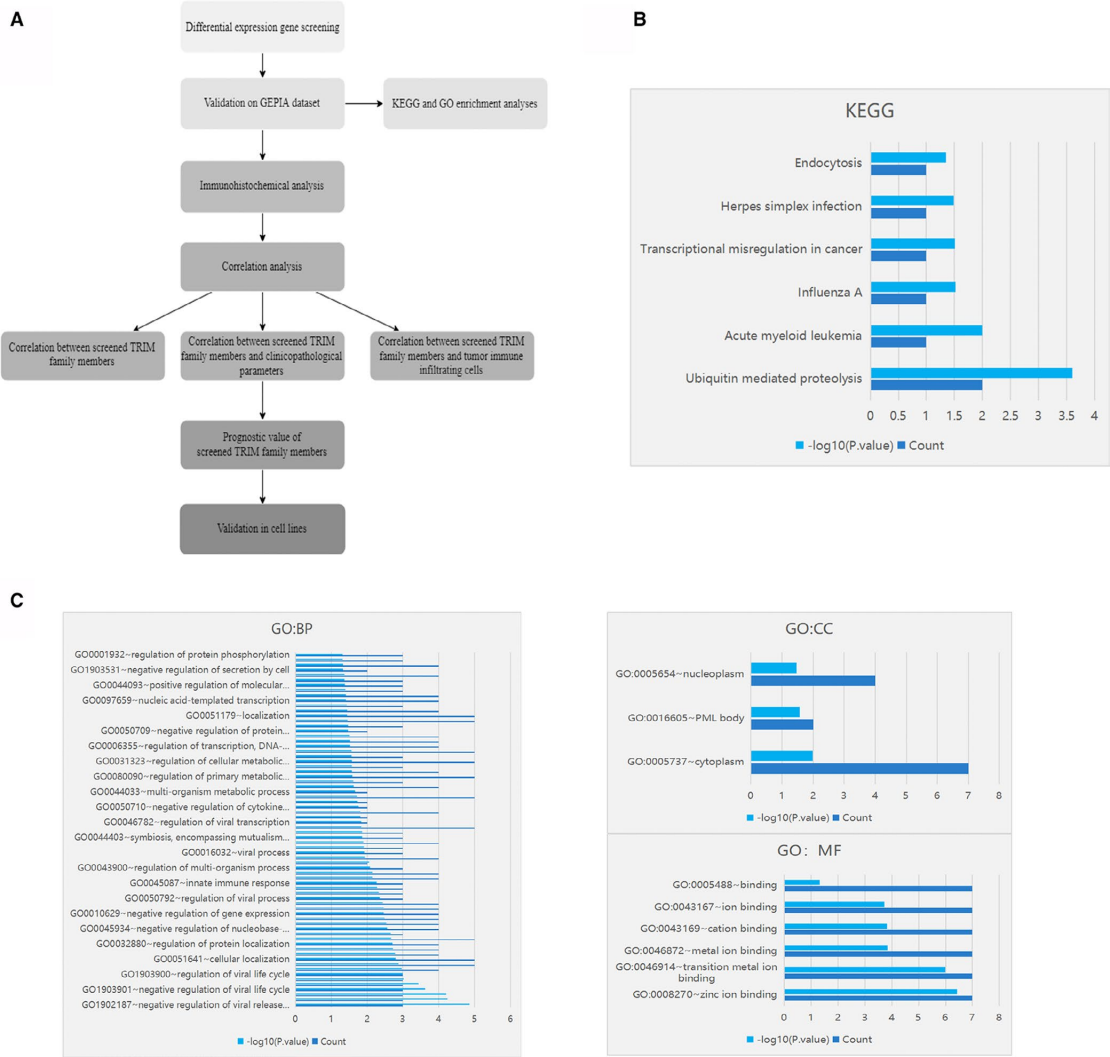
(A), Workflow of the present study. (B) By using the DAVID (Annotation, Visualization and Integration Discovery Database) tool, Gene Ontology (GO) analysis was performed on TRIMs. (C) The functions of TRIMs were predicted by analysis of Kyoto Encyclopedia of Genes and Genomes (KEGG) by DAVID (Database for Annotation, Visualization, and Integrated Discovery) tools

**FIGURE 2:**
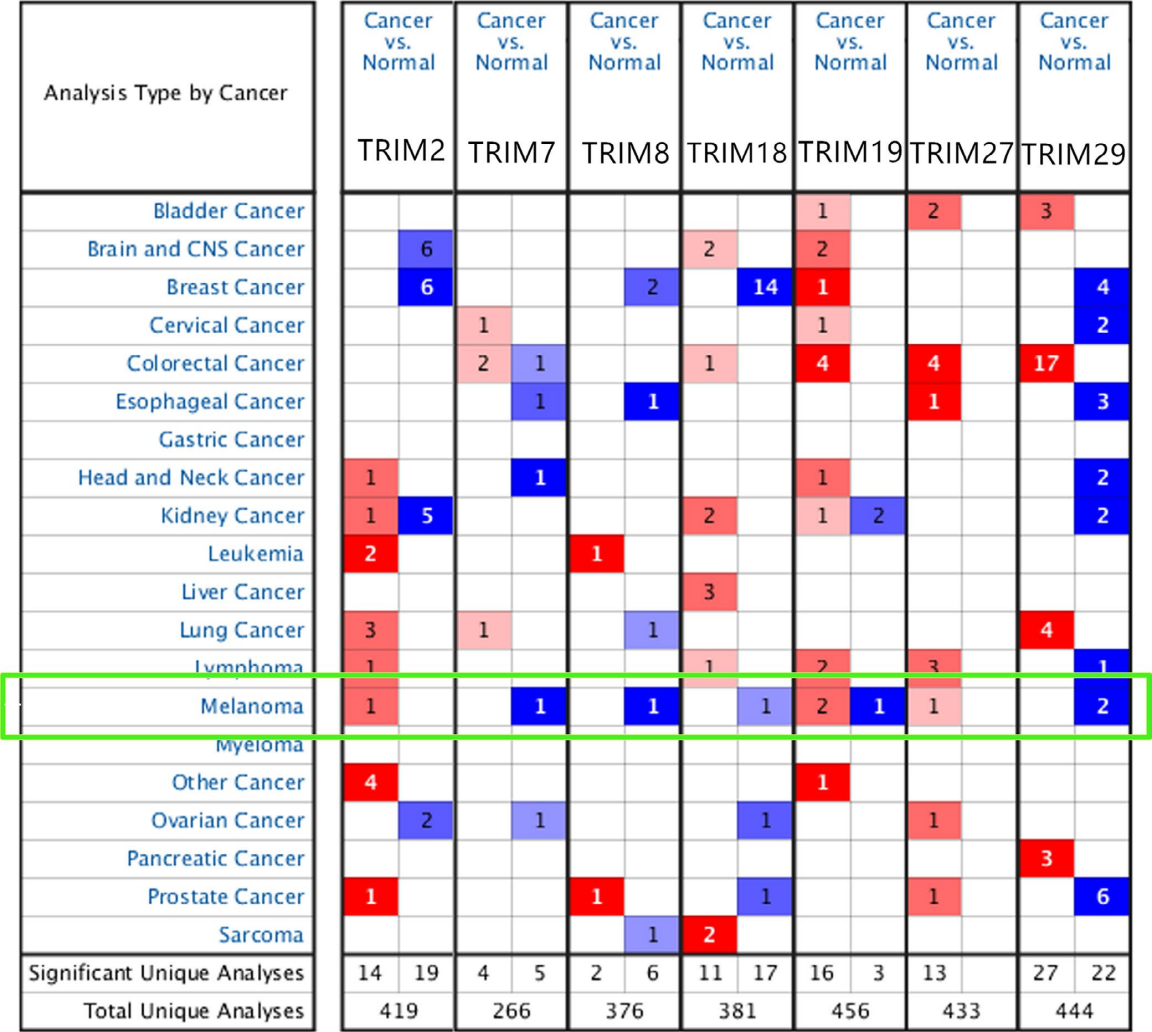
Transcriptional expression of TRIMs in 20 different types of cancer (Oncomine database). Differences in transcriptional expression were compared by Student's *t*‐test. The cut‐off values were as follows: *p* value: 1E‐4, fold change: 2, gene rank: 10%. Red represents high expression in tumor tissue and blue represents low expression

A flowchart of this study is revealed in Figure 1A. The gene expression of TRIM2 and TRIM27 was markedly higher, and the expression of TRIM7, TRIM8, and TRIM29 was significantly downregulated in melanoma. TRIM19 was expressed at low levels in some samples and upregulated in other samples (Figure 2).

**FIGURE 3:**
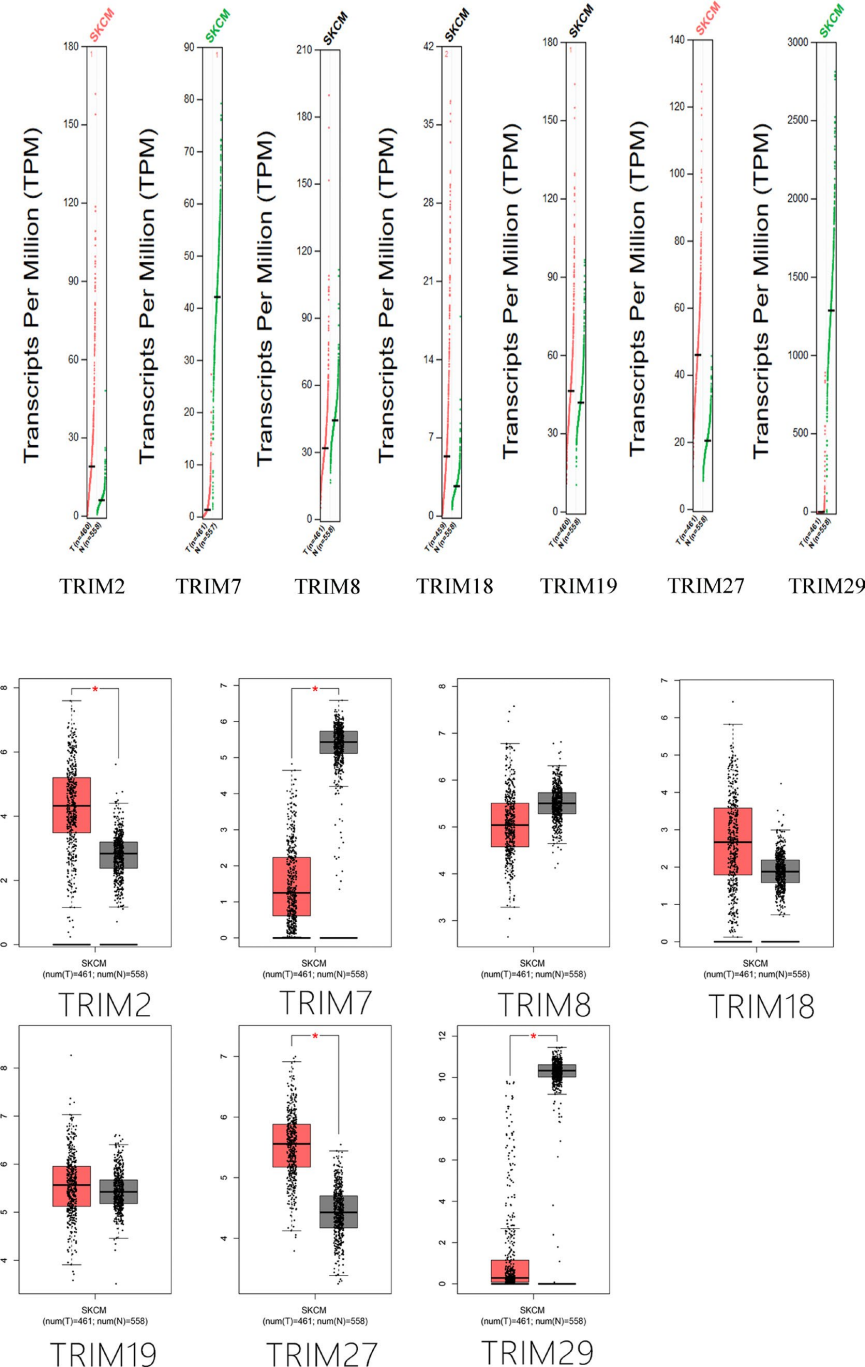
mRNA expression of different TRIM family members in melanoma tissue and adjacent normal tissues. Among them, TRIM2/27 was significantly overexpressed in melanoma, and TRIM7/29 was downregulated in tumor tissues

In the GEPIA database (Figure 3), TRIM2 and TRIM27 expression were significantly higher, and TRIM7 and TRIM29 expression was significantly downregulated in melanoma; these results were consistent with data from the Oncomine database (all *p* < 0.05). TRIM18 was slightly higher expressed in melanoma, but the difference was not statistically significant

In the DAVID database, the function of the screened TRIM family members was predicted (Figure 1B–C). In the analysis of cell components, the selected members of the TRIM family were mainly enriched in GO: 0005737 (cytoplasm), GO: 0016605 (PML body), and GO: 0005654 (nucleoplasm), and the molecular function was mainly enriched in zinc ion binding, which may be affected by the structure of the TRIM family members. Through KEGG analysis, the main enriched pathways were ubiquitin‐mediated proteolysis, acute myeloid leukemia, and influenza A; ubiquitination is one of the main mechanisms of the TRIM family involved in tumorigenesis.^29^, ^30^, ^31^, ^32^, ^33^, ^34^

### 3.1 Protein expression pattern of TRIMs in melanoma

**FIGURE 4:**
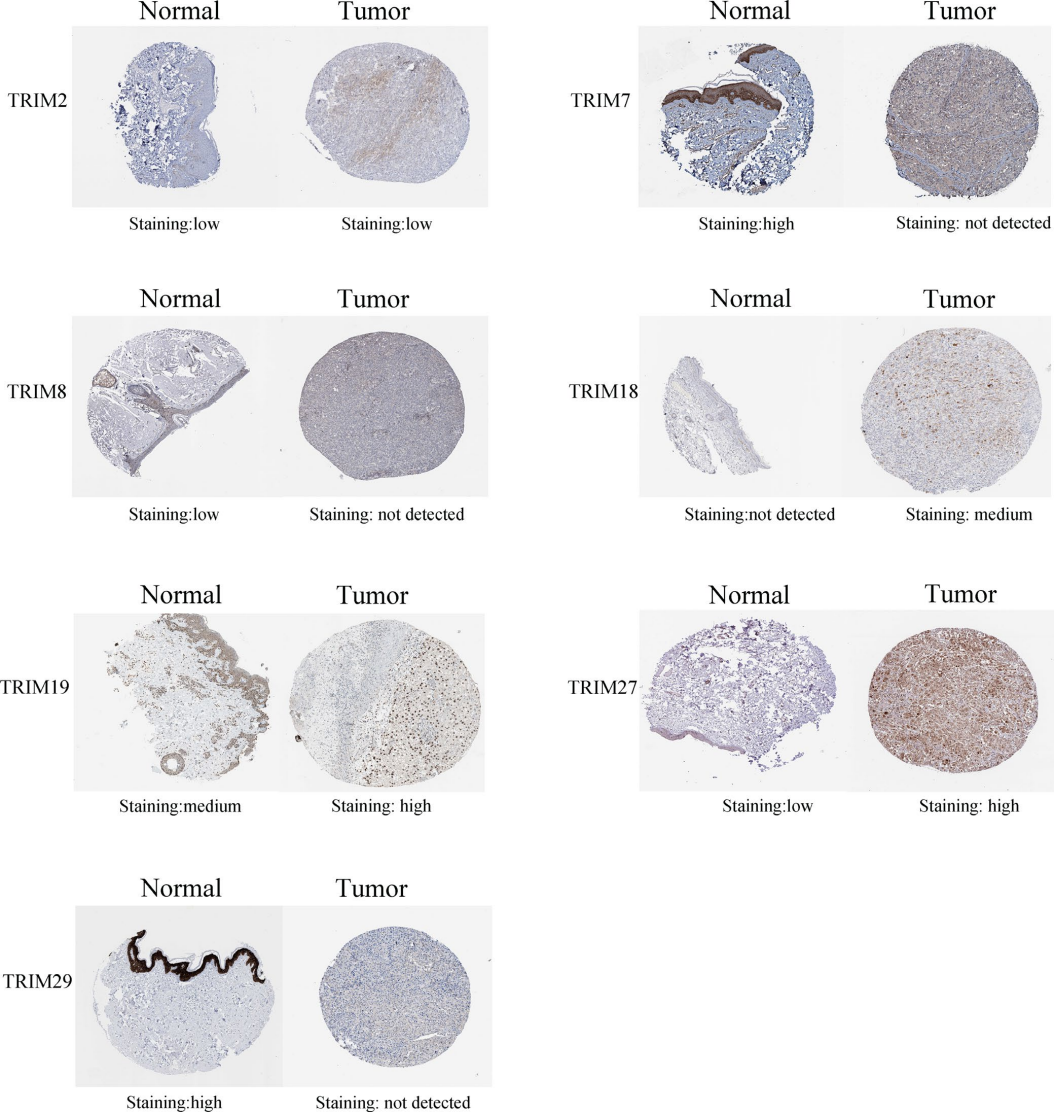
Representative immunohistochemical images of different TRIM family members in melanoma tissue and normal tissues. TRIM7 and TRIM29 were highly stained in normal tissues, but could not be detected in melanoma. TRIM27 was highly stained in melanoma and was up‐regulated at the protein level

The protein expression patterns of TRIMs in melanoma were explored by the Human Protein Atlas database (Figure 4). TRIM2 was faintly expressed in normal tissues and melanoma. TRIM7/8/29 were less frequently expressed in melanoma than in normal tissue. TRIM7 and TRIM29 were highly expressed in melanocytes in normal skin tissues but were not detectable in melanoma. TRIM18, TRIM19, and TRIM27 were upregulated in melanoma. TRIM27 was faintly expressed in cutaneous tissues and strongly stained in slices of melanoma.

### 3.2 Correction between TRIMs in melanoma

**FIGURE 5:**
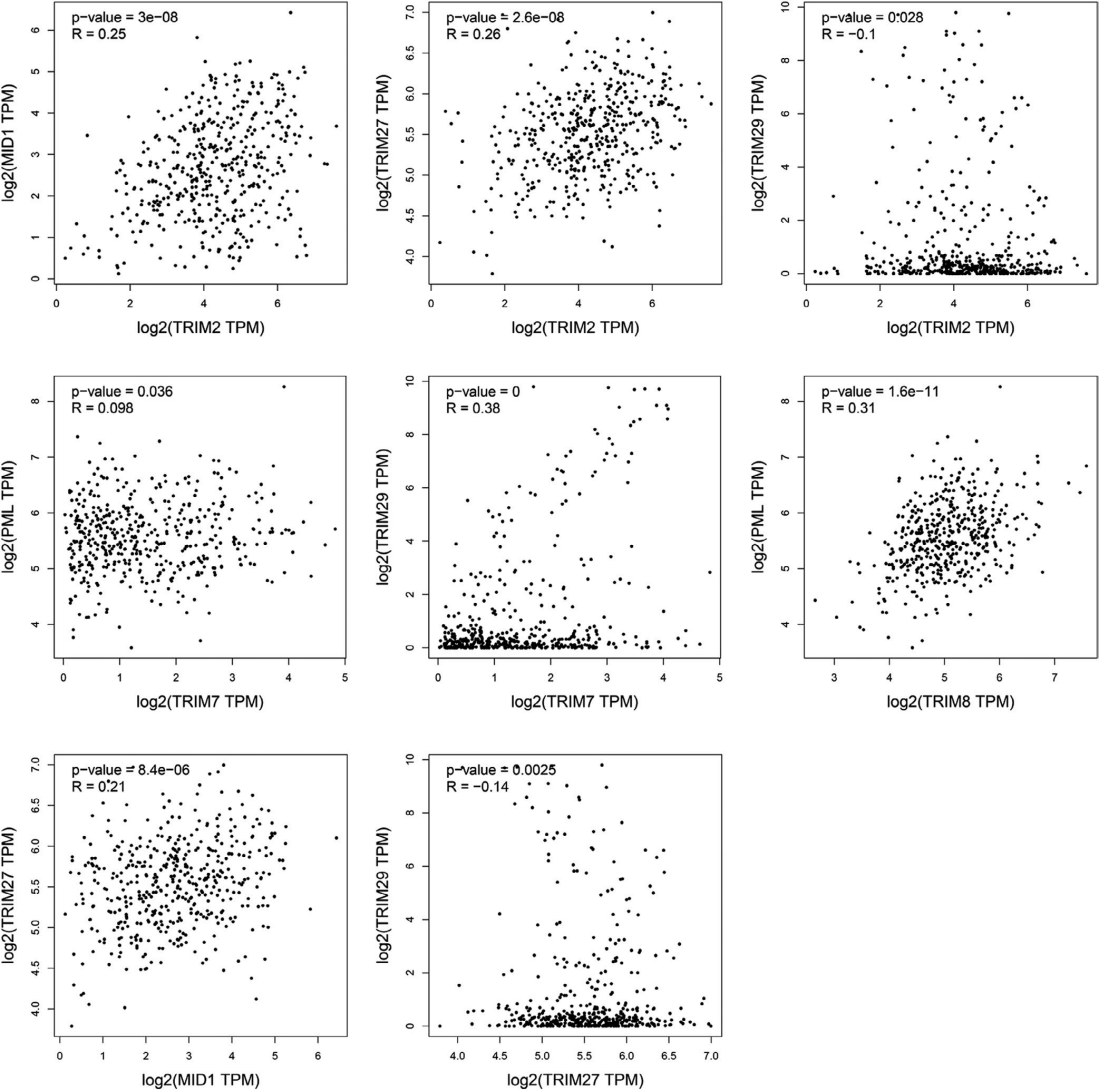
A statistically significant correlation between TRIM2, TRIM7, TRIM8, TRIM18, TRIM19, TRIM27, and TRIM29

We analyzed the correlations among TRIM2, TRIM7, TRIM8, TRIM18 (MID1), TRIM19 (PML), TRIM27, and TRIM29 by the GEPIA database. A total of 21 groups were analyzed, of which eight were statistically significant (Figure 5). TRIM2 was positively correlated with TRIM18 (MID1) (*R* = 0.25) and TRIM27 (*R* = 0.26) and negatively related to TRIM29 (*R* = −0.1). TRIM7 was bound up with TRIM19 (PML) (*R* = 0.098) and TRIM29 (*R* = 0.38). TRIM8 was positively correlated with TRIM19 (*R* = 0.31), TRIM18 was positively linked with TRIM27 (*R* = 0.21), and TRIM27 and TRIM29 were negatively correlated (*R* = −0.14).

### 3.3 Gene expression and clinical features

**FIGURE 6:**
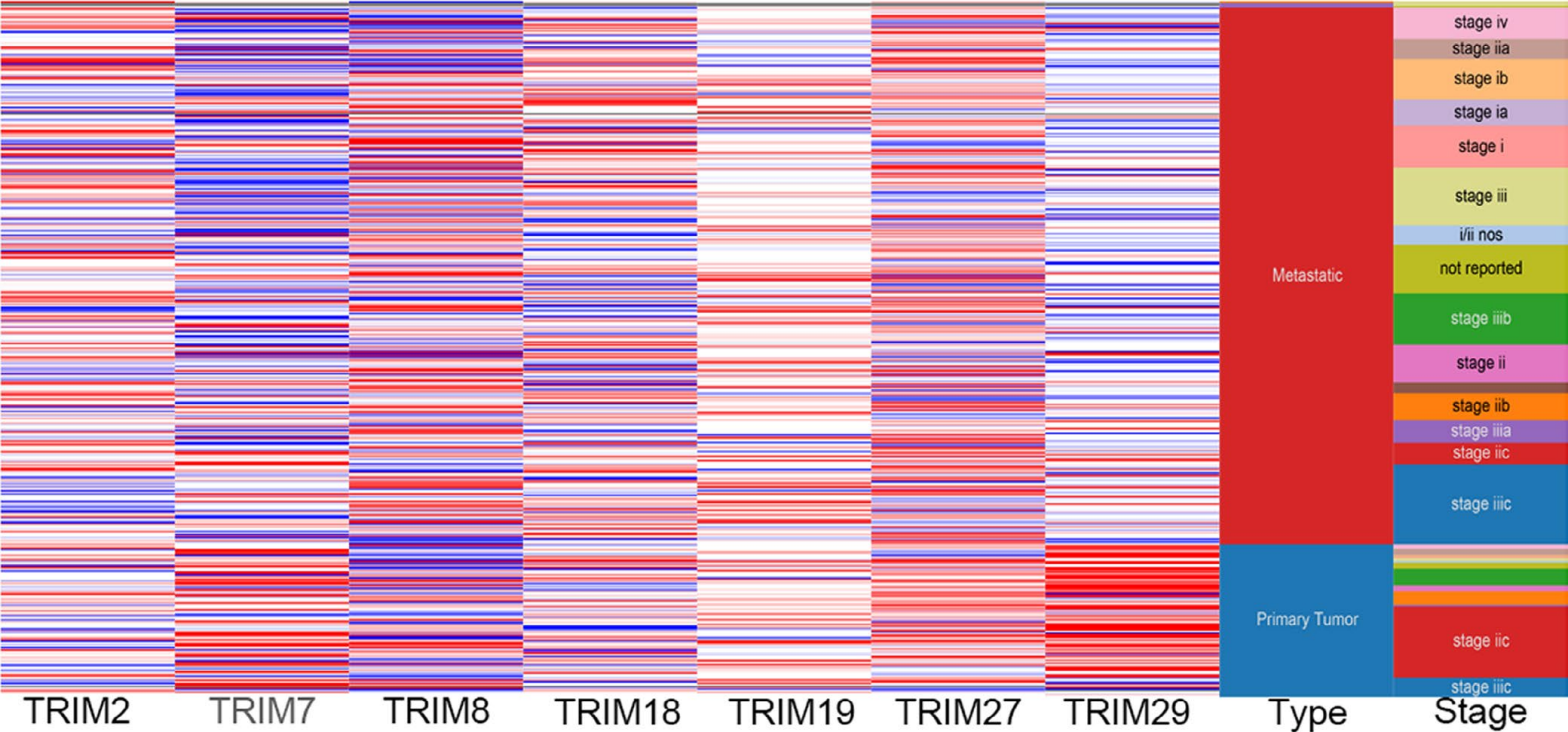
Hierarchical clustering of key genes was constructed using UCSC. Upregulation of genes is marked in red; downregulation of genes is marked in blue. TRIM7 and TRIM29 appeared to be mostly blue in metastatic melanoma, representing low expression, and mostly red in primary melanoma, representing high expression

In the UCSC database, we grouped tumor types and stages as indicators to observe the gene expression of TRIM family members (Figure 6). Among them, TRIM19 showed high expression in most tumor types and stages. Expression of TRIM7 and TRIM29 was mostly low in metastatic melanoma, as represented in blue in the figure, and mostly high in primary melanoma, represented in red.

### 3.4 TIICs and TRIM family

**FIGURE 7:**
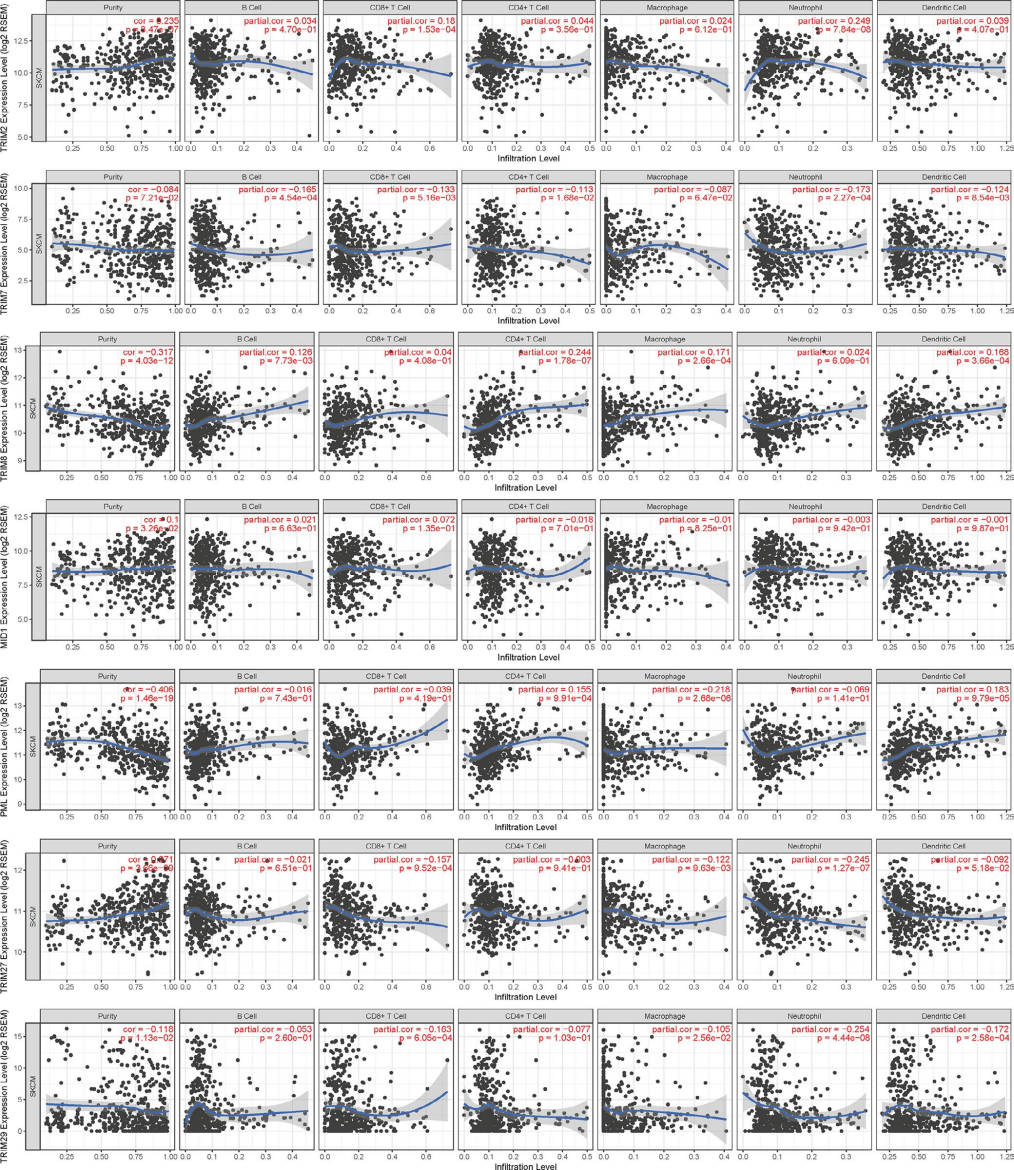
The correlation analysis between each type of tumor immune infiltrating cells (B‐cells, CD4^+^ T‐cells, CD8^+^ T‐cells, neutrophils, macrophages and dendritic cells) and TRIM family. There is no prominent relationship between TRIM family members and tumor immune infiltrating cells

The correlation between TRIMs and TIICs was not very prominent (Figure 7). Neutrophils were correlated with TRIM2 (correlation = 0.249, *p* = 7.84e‐8), TRIM27 (correlation = −0.245, *p* = 1.27e‐7), and TRIM29 (correlation = −0.254, *p* = 4.44e‐8). TRIM8 was related to CD4^+^ T cells (correlation = 0.244, *p* = 1.78e‐7). TRIM19 was related to macrophages (correlation = −0.218, *p* = 2.68e‐6).

### 3.5 Prognostic value of TRIMs in melanoma

**FIGURE 8:**
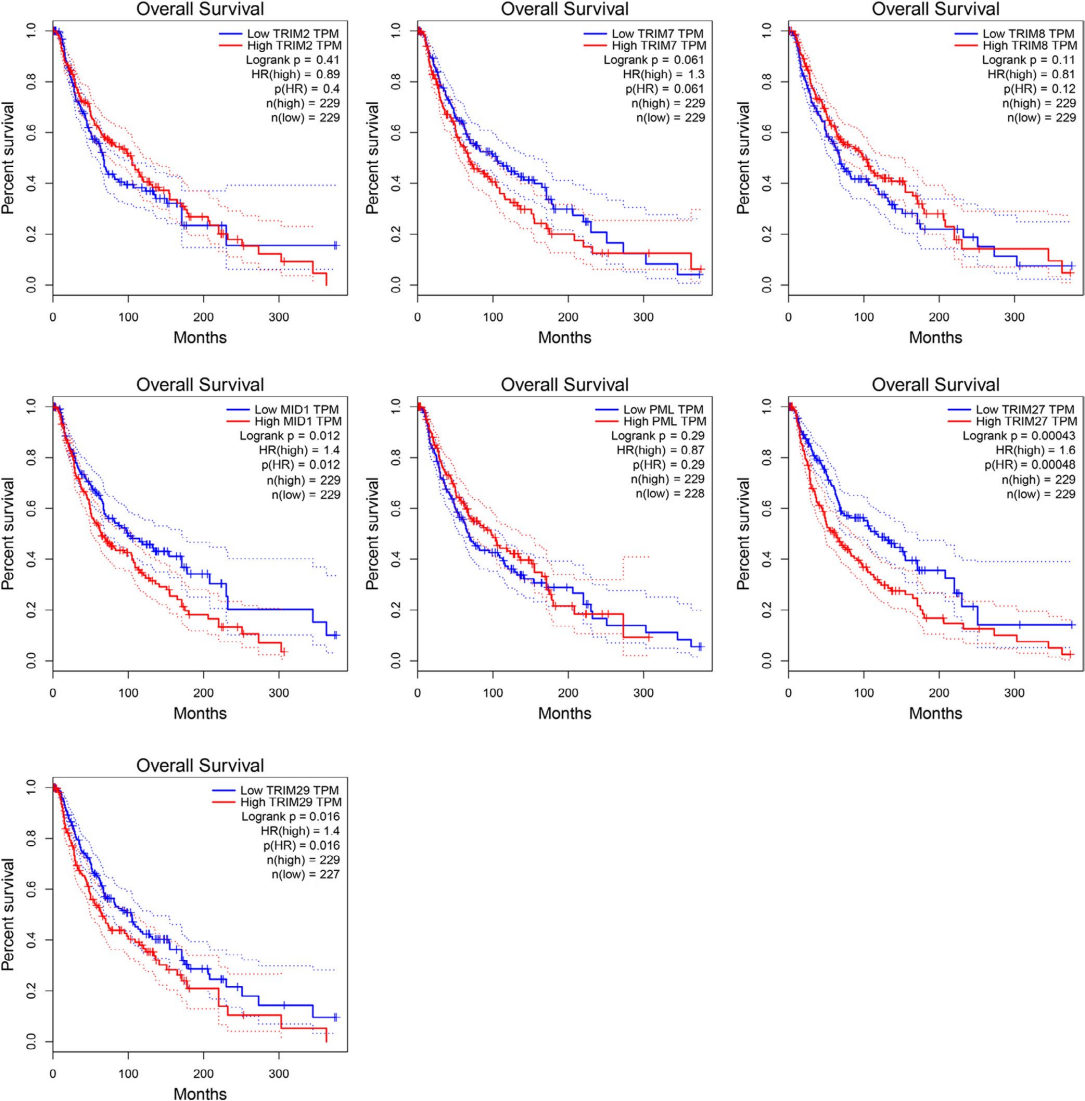
The overall survival analysis curve of low and high‐risk groups of the TRIMs signature. Among them, only the expression of TRIM18/27/29 had a significant effect on the overall survival of patients

**FIGURE 9:**
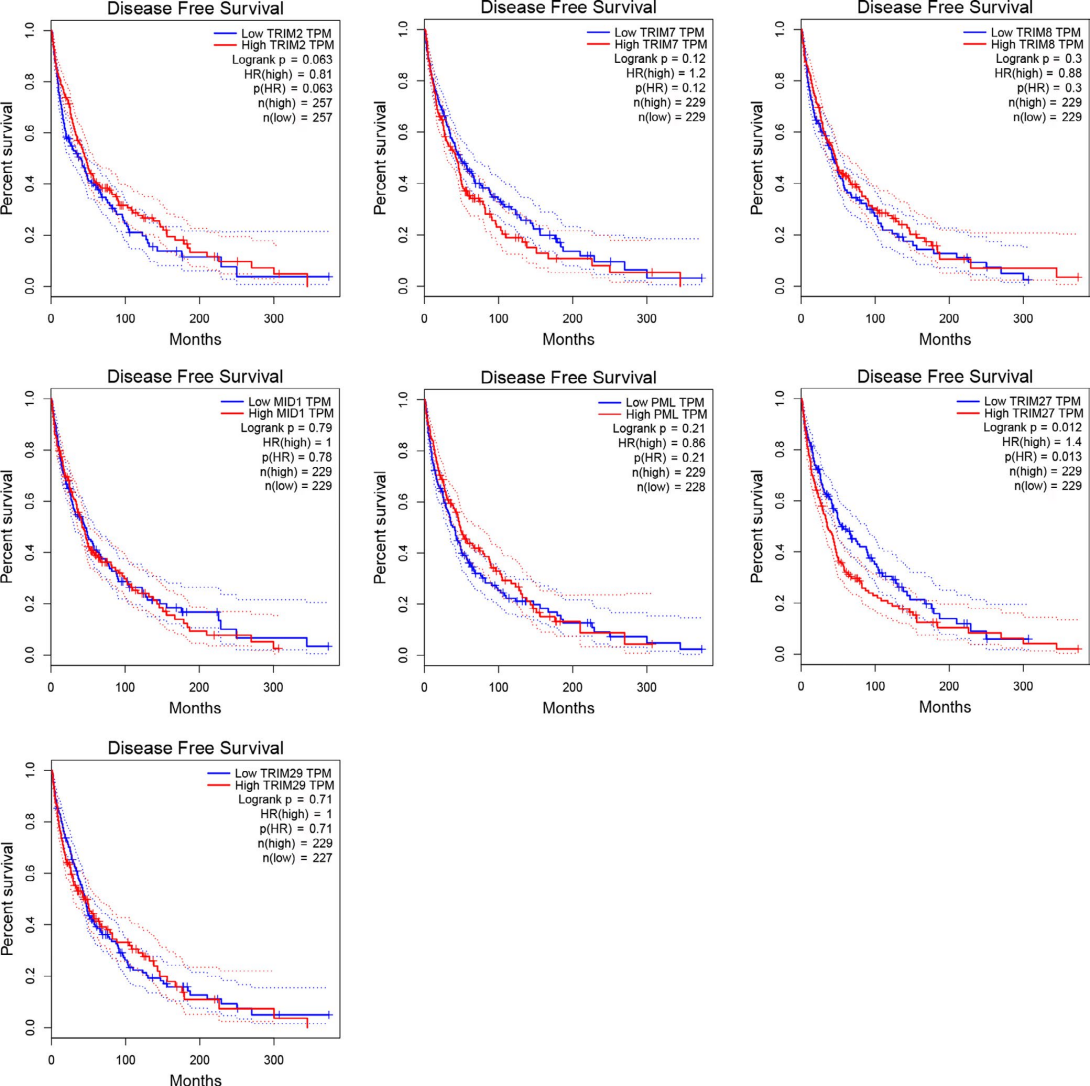
The disease‐free survival analysis curve of low and high‐risk groups of the TRIMs signature. Of the seven possible key genes, only TRIM27 was significantly associated with disease‐free survival in melanoma patients

We used the GEPIA database to investigate the prognostic analysis of TRIM2, TRIM7, TRIM8, TRIM18 (MID1), TRIM19 (PML), TRIM27, and TRIM29 in melanoma. The results suggested that TRIM18‐ (*p* = 0.012), TRIM27‐ (*p* = 0.00043), and TRIM29‐ high expression (*p* = 0.016) were significantly linked with shorter survival time. Highly expressed TRIM18, TRIM27, and TRIM29 were prognostic factors for overall survival (Figure 8). Only TRIM27 significantly affected DFS, and patients with low expression of TRIM27 had better DFS (*p* = 0.012). TRIM18 and TRIM29 had no significant effect on DFS (Figure 9).

In the univariate analysis, the hazard ratio (HR) of TRIM27 and TRIM29 was 1.351 and 1.070, respectively, which were significantly bound up with patient survival. There is a prominent relevancy between TRIM27 (HR = 1.577, 95% CI 1.054–2.606, *p* = 0.026）and the increased risk of survival in multivariable analysis (Table 2).

**TABLE 2:** The effects of different TRIM expression and clinicopathological characteristics on the overall survival of patients were implemented through univariate and multivariate Cox hazards regression analysis

Variables	Univariable		Multivariable	
	HR (95% CI)	*p* value	HR (95% CI)	*p* value
TRIM2	0.939 (0.842–1.049)	0.265	0.788 (0.647–1.061)	0.068
TRIM7	0.818 (0.332–2.017)	0.662	1.059 (0.388–2.890)	0.911
TRIM8	0.993 (0.826–1.193)	0.939	0.861 (0.567–1.309)	0.485
TRIM18	1.041 (0.901–1.203)	0.584	1.131 (0.922–1.386)	0.237
TRIM19	1.051 (0.847–1.305)	0.652	0.927 (0.638–1.348)	0.691
TRIM27	1.351 (1.033–1.640)	0.037	1.577 (1.054–2.606)	0.026
TRIM29	1.070 (1.002–1.142)	0.044	1.053 (0.979–1.132)	0.164
Age				
0–40	Reference		Reference	
40–60	1.691 (0.775–3.694)	0.187	1.255 (0.503–3.127)	0.627
60–80	1.215 (0.259–5.701)	0.805	0.555 (0.101–3.050)	0.498
>80	—	—	—	—
Gender				
Male	Reference		Reference	
Female	0.837 (0.496–1.411)	0.504	0.648 (0.363–1.157)	0.142
Race				
African American	Reference		Reference	
Asian	0.902 (0.124–6.543)	0.918	0.741 (0.090–6.112)	0.781
White	9.123 (3.715–22.401)	<0.001	6.443 (2.026–20.490)	0.002
Sample type				
Metastatic	Reference		Reference	
Primary tumor	0.441 (0.221–0.880)	0.020	0.972 (0.400–2.361)	0.950
Tumor stage				
I	Reference		Reference	
II	2.377 (0.755–7.484)	0.139	1.590 (0.432–5.855)	0.486
III	3.400 (1.072–10.777)	0.038	4.487 (0.880–22.874)	0.071
IV	5.141 (1.171–22.578)	0.030	0.980 (0.164–5.869)	0.982
Breslow depth (mm)				
0–1.0	Reference		Reference	
1.1–2.0	2.820 (0.705–11.290)	0.143	1.709 (0.297–9.826)	0.548
2.1–3.0	6.671 (1.713–25.972)	0.006	2.694 (0.462–15.714)	0.271
3.1–4.0	7.004 (2.077–23.621)	0.002	3.379 (0.626–18.231)	0.157
>4.0	2.693 (0.763–9.504)	0.124	1.413 (0.227–8.790)	0.711
Distant metastasis				
No/unknown	Reference		Reference	
Yes	0.739 (0.334–1.637)	0.456	0.709 (0.282–1.780)	0.464
Ulceration				
No	Reference		Reference	
Yes	3.031 (1.609–5.710)	0.001	1.384 (0.615–3.115)	0.432
Unknown	1.070 (0.535–2.138)	0.849	1.370 (0.527–3.562)	0.519
Systemic therapy				
No	Reference		Reference	
Yes	0.354 (0.086–1.451)	0.149	0.350 (0.078–1.557)	0.168
Radiation therapy				
No	Reference		Reference	
Yes	0.330 (0.119–0.913)	0.033	0.470 (0.161–1.370)	0.167
Unknown	0.246 (0.060–1.012)	0.052	0.263 (0.058–1.185)	0.082

Abbreviations: CI, confidence interval; HR, hazard ratio.

### 3.6 Validation of the screened genes by qRT‐PCR

**FIGURE 10:**
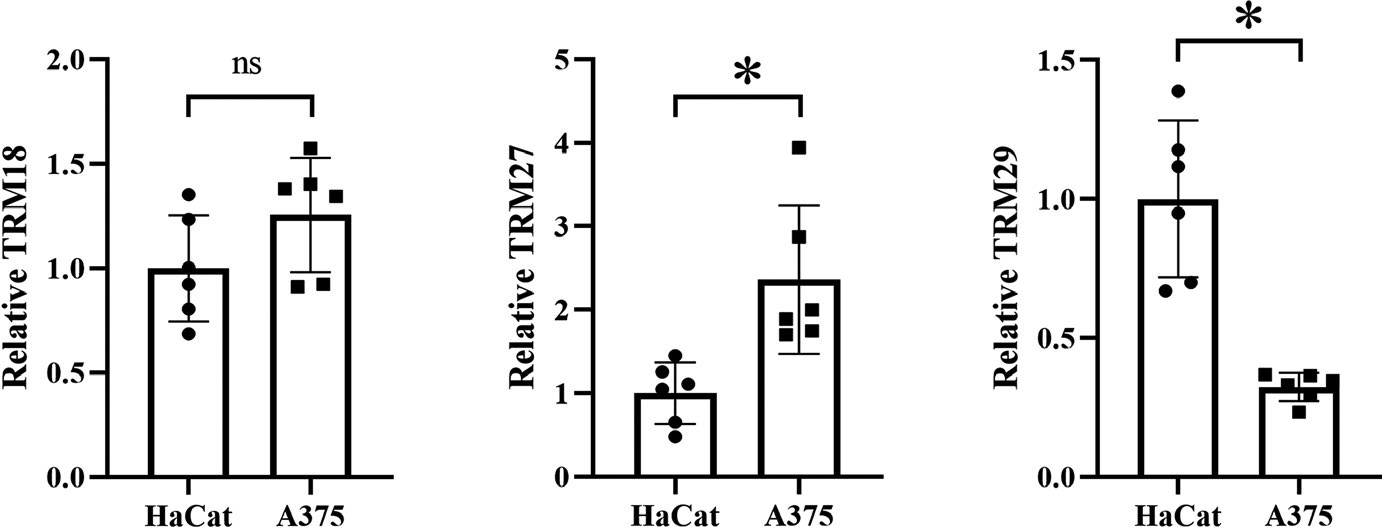
Expression of TRIMs that have a significant impact on survival (TRIM18/27/29) in melanoma cell line A375 compared to the normal cell line. “*” means statistically significant

This study verified the expression of TRIMs with a significant impact on survival by qRT‐PCR methods in melanoma cell line. The results showed that compared with normal lines, TRIM27 was highly expressed in melanoma cell line A375, and TRIM29 was downregulated in melanoma cell line A375 (Figure 10), and both had statistical significance (*p* < 0.05). After verification by qRT‐PCR, TRIM18 was highly expressed in melanoma cell lines A375, but the difference was not statistically significant.

### 3.7 Gene‐set enrichment analysis of TRIM27

**FIGURE 11:**
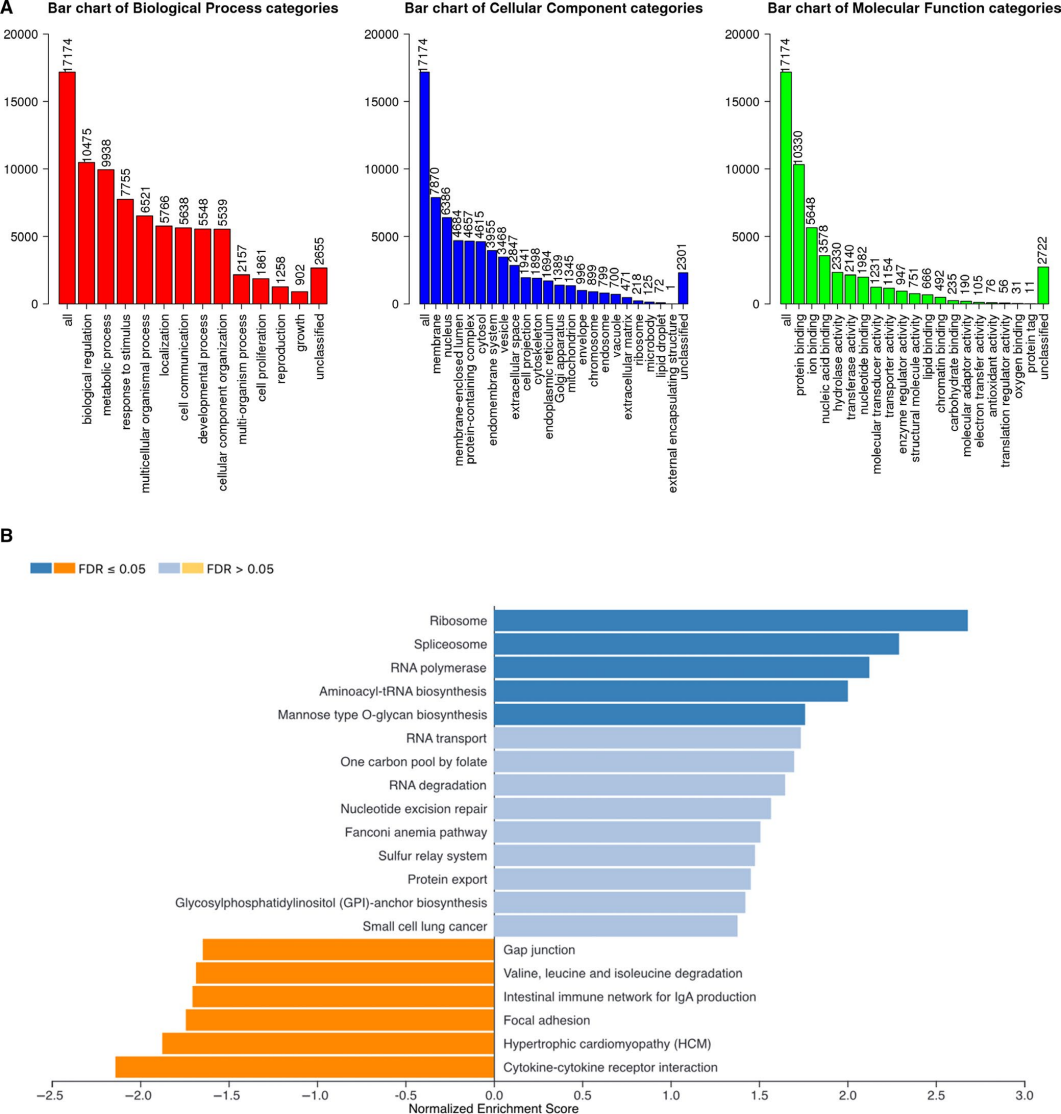
Gene Ontology (GO) analysis and Kyoto Encyclopedia of Genes and Genomes (KEGG) analysis of the TRIM27 gene in melanoma. (A) GO analysis. (B) KEGG analysis

In GO analysis (Figure 11A), TRIM27 was mainly involved in biological regulation, metabolic processes, membranes, the nucleus, protein binding, and ion binding. In KEGG analysis (Figure 11B), TRIM27 is mainly involved in ribosome and cytokine‐cytokine receptor interaction. The main biological processes and molecular functions involved are shown in Table 3.

**TABLE 3:** Enriched GO and KEGG items

Enriched category	Description	Count	NES	*p*‐value	FDR
Biological process					
GO:0034470	ncRNA processing	131	2.6397	0	0
GO:0022613	ribonucleoprotein complex biogenesis	142	2.6115	0	0
GO:0006414	Translational elongation	51	2.4177	0	0
GO:0140053	Mitochondrial gene expression	61	2.3932	0	0
GO:0016072	rRNA metabolic process	101	2.3769	0	0
GO:0071826	Ribonucleoprotein complex subunit organization	74	2.3382	0	0
GO:0006353	DNA‐templated transcription, termination	20	2.3211	0	0
GO:0008380	RNA splicing	131	2.3051	0	0
GO:0006397	mRNA processing	138	2.2775	0	0
GO:0070972	Protein localization to endoplasmic reticulum	85	2.2653	0	0
GO:0006399	tRNA metabolic process	62	2.2373	0	0
Cellular components					
GO:0005840	Ribosome	117	2.6672	0	0
GO:0098798	Mitochondrial protein complex	76	2.3294	0	0
GO:0030684	Preribosome	38	2.2291	0	0
GO:0005681	Spliceosomal complex	57	2.1876	0	0
GO:0098636	Protein complex involved in cell adhesion	25	−1.9222	0	0
GO:0031012	Extracellular matrix	191	−2.0041	0	0
Molecular function					
GO:0003735	Structural constituent of ribosome	95	2.6895	0	0
GO:0140098	Catalytic activity, acting on RNA	94	2.3117	0	0
GO:0019843	rRNA binding	30	2.2519	0	0
GO:0050840	Extracellular matrix binding	32	−2.0991	0	0
GO:0019955	Cytokine binding	79	−2.1061	0	0
KEGG pathway					
hsa03010	Ribosome	98	2.6816	0	0
hsa03040	Spliceosome	34	2.2925	0	0
hsa04640	Hematopoietic cell lineage	52	−2.0262	0	0
hsa04060	Cytokine‐cytokine receptor interaction	149	−2.1399	0	0
hsa04512	ECM‐receptor interaction	48	−2.1555	0	0

Note

Table shows three items each from GO‐BP, GO‐CC, GO‐MF, and KEGG.

## 4 DISCUSSION

Although many scholars have studied the TRIM family in cancer, the TRIM family has rarely been studied in melanoma. Our results indicated differential expression of TRIM2, TRIM7, TRIM8, TRIM18 (MID1), TRIM19 (PML), TRIM27, and TRIM29. The relationship between the selected TRIMs and TIICs was not very prominent. The current literature has confirmed the relationship between TRIM33 and TRIM72, but other TRIMs have not been reported. It has been confirmed that TRIM33 is involved in the production and activation of macrophages^35^ and is a vital transcription regulator for macrophage/monocyte‐mediated inflammation; TRIM72 regulates alveolar macrophages to participate in lung innate immunity.^36^ Three genes (TRIM18/27/29) were significant in prognostic analysis of overall survival. In terms of DFS, only TRIM27 had a correlation with survival, and its expression was confirmed by qRT‐PCR. Therefore, TRIM27 may be a prognostic biomarker for melanoma. TRIM29 appeared to have low expression in metastatic melanoma and high expression in primary melanoma. The role of TRIM29 in melanoma still needs further experimental verification. Studies on TRIM29 and melanoma have not yet been found, which may be a good research direction.

Most TRIM family members are considered as E3 ubiquitin ligases because of the structure of the zinc‐finger domain.^5^, ^7^, ^37^, ^38^, ^39^ TRIM family is part of multifarious physiological processes, such as DNA damage repair, intracellular signal transduction, and immune responses. The most important cell cycle phase involving TRIMs is mitosis. TRIMs abnormal expression and possible mechanisms have been proved in diversiform cancers, but this is the first time that TRIMs were explored in melanoma. We hope that this article will help improve treatment design and improve the prognosis of melanoma patients.^9^, ^32^, ^40^, ^41^

Overexpression of the TRIM family member TRIM2 promotes the migration, invasion, and proliferation of colorectal cancer, and Cao et al. demonstrated that TRIM2 may be a new biomarker for colorectal cancer.^13^ Ras/MAPK signaling affects cell proliferation and apoptosis by regulating c‐Jun/AP‐1 transcription factors. Chakraborty et al. demonstrated that TRIM7 activated AP‐1 via RAS and validated the meaning of the TRIM7 in tumorigenesis.^42^

In the development of cancer, TRIM8 seems to have a dual role. TRIM8 exerts a suppressor effect by actively affecting the NF‐κB pathway. On the other hand, TRIM8 exerts an oncogenic effect by regulating p53 tumor suppressor activity.^43^, ^44^

TRIM18, also known as midline 1 (MID1), encodes a protein that forms a homodimer with tubulin in the cytoplasm.^45^ TRIM19 (PML) is associated with the occurrence of promyelocytic leukemia, participates in cell cycle, and responds to P53 oncogene signals.^46^

TRIM27 was found to be involved in the fusion and recombination of proto‐oncogenes, and can regulate cell cycle and induce apoptosis by up‐regulating p‐P38 expression and down‐regulating P‐Akt expression.^47^ TRIM29 can inhibit or activate the progression of hepatocellular carcinoma and colorectal cancer by targeting Wnt/β‐catenin signaling pathway.^17^

As a comprehensive bioinformatics analysis study, this article had some limitations. First, the possible mechanism of action for TRIM27 and related pathways has not been verified in in vivo or in vitro molecular biology experiments. Further biological experiments are our future research directions. Second, bioinformatics analysis using different tools and different databases may yield different results; therefore, our results were not very comprehensive, and there may be many other genes that are differentially expressed in melanoma waiting to be discovered and verified. Since this article only collected the expression of TRIMs in the Oncomine database; thus, there is a certain bias, and it is necessary to verify TRIMs in more databases, cell lines, and clinical samples. Third, we only validated the expression of TRIMs at the molecular level, and more patients need to be included in the study to verify their feasibility biomarkers.

## 5 CONCLUSION

By various data sets and comprehensive methods, we not only identified TRIMs that are differentially expressed in melanoma but also discovered that TRIM27 may be considered a new potential biomarker for melanoma. Therefore, our results provide a novel biomarker for melanoma, which may be helpful for the determining the prognosis of melanoma and further clinical application of targeted therapy.

## CONFLICT OF INTEREST

The authors declare that they have no competing interests.

## AUTHORS’ CONTRIBUTIONS

YCJ conceived and designed the study. XYJ, ZJ, and YCJ wrote the manuscript. XYJ, ZJ, and YCJ performed the data analysis and revised the manuscript critically. XYJ and ZJ contribute equally to this article.

## COMPLIANCE WITH ETHICAL STANDARDS

This article does not contain any studies with human participants or animals performed by any of the authors.

## INFORMED CONSENT

Not applicable.

## Data Availability

All data were generated or analyzed during this study are included in this published article.
